# Reply to Dr. Takeuchi: PRES after epidural anesthesia

**DOI:** 10.1007/s10194-011-0343-3

**Published:** 2011-05-01

**Authors:** Silvia Pugliese, Alessandro Bozzao

**Affiliations:** Department of Neuroradiology, University of Rome La Sapienza , Rome, Italy

We thank Dr Satoru Takeuchi and his colleagues for their comments [1] about our article on the probable causal association between intracranial hypotension (IH) and posterior reversible encephalopathy syndrome (PRES) [2]. They ask us to further debate the possible role of reversible cerebral vasoconstriction syndrome (RCVS) in the clinical history of our patient.

RCVS is a group of disorders characterized by the reversible segmental and the multifocal vasoconstriction of cerebral arteries at angiography and severe “thunderclap” headache with or without focal neurological deficits or seizures [3].

In our patient, RCVS was mainly ruled out because of normal MRA findings. As described by Ducros et al. [4], some patients (only 9% of the cases) even with an initial normal MRA could have a repeated MRA showing vessels narrowing, but we did not suspected RCVS because of the headache clinical features and the neurological signs. In fact, our patient presented a bilateral headache, pressure-like, with a postural component, different from the thunderclap headache, typical of RCVS. It is possible that the mild headache onset was consequent to the epidural anesthesia, but the headache did not change in severity until the patient discharge, some days after the end of anesthesia effect. Moreover, the MR findings were typical of PRES, showing multiple symmetric and bilateral hypertintensities on T2-weighted images in the posterior territories, with high signal on DWI, as well as on ADC maps. It has been described that a post-partum RCVS could cause PRES [5, 6], but we supposed that the demonstrated intra-cranial hypotension (IH), subsequent to the inadvertent dural puncture, was the leading cause of PRES. This hypothesis is mainly supported by the prompt resolution of the neurological symptoms and the radiological alterations of both IH and PRES, only after the treatment of IH with a blood patch. This would be difficult to be expected in case of RCVS.

Finally, we know that the association between IH and PRES is only a presumption and that there are some limitations of our single experience and of the proofs provided. We look forward to further contributions that could eventually clarify the relationship between IH and PRES.
